# A population of CD4^hi^CD38^hi^ T cells correlates with disease severity in patients with acute malaria

**DOI:** 10.1002/cti2.1209

**Published:** 2020-11-24

**Authors:** Simon H Apte, Gabriela Minigo, Penny L Groves, Jessie C Spargo, Magdalena Plebanski, Mathew J Grigg, Enny Kenangalem, Julie G Burel, Jessica R Loughland, Katie L Flanagan, Kim A Piera, Timothy William, Ric N Price, Tonia Woodberry, Bridget E Barber, Nicholas M Anstey, Denise L Doolan

**Affiliations:** ^1^ Infectious Diseases Program QIMR Berghofer Medical Research Institute Brisbane QLD Australia; ^2^ Global and Tropical Health Division Menzies School of Health Research Casuarina NT Australia; ^3^ Charles Darwin University Darwin NT Australia; ^4^ Department of Immunology and Pathology Monash University Prahran VIC Australia; ^5^ School of Health and Biomedical Sciences RMIT University Bundoora VIC Australia; ^6^ Papuan Health and Community Development Foundation Timika Indonesia; ^7^ School of Medicine University of Tasmania Launceston TAS Australia; ^8^ Nuffield Department of Clinical Medicine Centre for Tropical Medicine and Global Health University of Oxford Oxford UK; ^9^ Mahidol‐Oxford Tropical Medicine Research Unit Faculty of Tropical Medicine Mahidol University Bangkok Thailand; ^10^ Centre for Molecular Therapeutics Australian Institute of Tropical Health & Medicine James Cook University Cairns QLD Australia; ^11^ Present address: Queensland Lung Transplant Service, The Prince Charles Hospital Chermside QLD Australia; ^12^ Present address: La Jolla Institute for Immunology La Jolla CA USA

**Keywords:** CD4^+^ T cells, regulatory T cells, malaria, CD4 co‐receptor modulation, CD38

## Abstract

**Objective:**

CD4^+^ T cells are critical mediators of immunity to *Plasmodium* spp. infection, but their characteristics during malarial episodes and immunopathology in naturally infected adults are poorly defined. Flow cytometric analysis of PBMCs from patients with either *P. falciparum* or *P. knowlesi* malaria revealed a pronounced population of CD4^+^ T cells co‐expressing very high levels of CD4 and CD38 we have termed CD4^hi^CD38^hi^ T cells. We set out to gain insight into the function of these novel cells.

**Methods:**

CD4^+^ T cells from 18 patients with *P. falciparum* or *P. knowlesi* malaria were assessed by flow cytometry and sorted into populations of CD4^hi^CD38^hi^ or CD4^norm^ T cells. Gene expression in the sorted populations was assessed by qPCR and NanoString.

**Results:**

CD4^hi^CD38^hi^ T cells expressed high levels of *CD4* mRNA and canonical type 1 regulatory T‐cell (TR1) genes including *IL10*, *IFNG*, *LAG3* and *HAVCR2* (TIM3), and other genes with relevance to cell migration and immunomodulation. These cells increased in proportion to malaria disease severity and were absent after parasite clearance with antimalarials.

**Conclusion:**

In naturally infected adults with acute malaria, a prominent population of type 1 regulatory T cells arises that can be defined by high co‐expression of CD4 and CD38 (CD4^hi^CD38^hi^) and that correlates with disease severity in patients with falciparum malaria. This study provides fundamental insights into T‐cell biology, including the first evidence that CD4 expression is modulated at the mRNA level. These findings have important implications for understanding the balance between immunity and immunopathology during malaria.

## Graphical abstract

In this study, we identified a population of CD4^+^ T cells that expresses very high levels of CD4 and CD38 (CD4^hi^CD38^hi^) in adult malaria patients living in malaria‐endemic regions. These cells were increased in proportion to disease severity, and gene expression analysis revealed them to be type 1 regulatory T cells. Increased CD4 co‐receptor surface expression by CD4^hi^CD38^hi^ T cells was modulated at the mRNA level revealing a level of commitment to the phenotype and a hitherto unseen mechanism by which CD4 T cells may potentially modulate their activity.

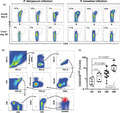

## Introduction

Malaria remains a significant threat to global health with 228 million cases and 405 000 deaths estimated in 2018^1^. The causative agent, the *Plasmodium* spp. parasite, has a complex multistage life cycle that has co‐evolved with the human host. The human immune response to the parasite is complex and engages innate and adaptive cellular and humoral immune components, and the parasite has developed multiple strategies to avoid the host immune response in order to persist. Managing the balance between controlling a rapidly multiplying and potentially fatal pathogen and avoiding immunopathology is difficult (reviewed in Coban *et al*.^2^, ^3^). Because of the complexity of this parasite–host relationship, immune correlates of protection remain unknown despite many decades of intensive research, and the development of an effective vaccine has proved elusive.

The blood stages of the *Plasmodium* spp. parasite life cycle are responsible for the clinical symptoms of malaria. Animal studies have demonstrated a critical role for CD4^+^ T cells in the control of the blood stage of *Plasmodium* infection (reviewed in Kurup *et al*.^4^), and immunisation studies utilising controlled human malaria infection (CHMI) of malaria‐naïve human volunteers have associated protection from reinfection with CD4^+^ T‐cell responses^5^, ^6^. Furthermore, mounting evidence in humans and animal models suggests that CD4^+^ T cells perform a critical cytokine‐mediated balancing act in order to mediate immunity without pathology, in particular, via production of pro‐inflammatory cytokines including IFN‐γ and TNF‐α, and the immunosuppressive cytokine IL‐10 (reviewed in Kumar *et al*.^7^).

In CHMI studies, we have shown that the control of the parasite in malaria‐naïve volunteers following the infection with *P. falciparum*‐infected red blood cells (pRBCs) is associated with the expansion of a population of CD4^+^ T cells co‐expressing the activation marker CD38^8^. However, in naturally exposed individuals from Uganda, an increased frequency of CD38‐expressing CD4^+^ T cells predicts treatment failure^9^, and in another study, CD38^+^ CD4^+^ T‐cell frequency correlated positively with HIV viral load, which was further accentuated in patients co‐infected with malaria^10^.

Although CD38 expression has primarily been used as a marker of CD4^+^ T‐cell activation, the CD38 molecule itself performs a multitude of functions and is ubiquitously expressed in different tissues. In broad terms, CD38 has three known functions in T cells: (1) it is a multifunctional ectoenzyme with activities that include catalysis of extracellular nicotinamide adenine dinucleotide (NAD), thereby regulating extracellular levels of NAD and modulating intracellular Ca^2+^ levels (reviewed in Malavasi *et al*.^11^); (2) it acts as a cellular receptor, associating with the T‐cell receptor (TCR) on lipid rafts on T cells, and transduces activation signals by phosphorylating tyrosine residues on CD3, ZAP‐70 and LAT^11^; and (3) it acts as a ligand, capable of binding CD31 on endothelial cells and participating in lymphocyte homing and inducing signalling through CD31 on CD31‐expressing cells^11^. These functions have particular relevance in the case of *Plasmodium* infection: infected RBCs have very high levels of NAD^12^, and lysis of infected RBC releases NAD into the extracellular milieu, which acts as an immune 'danger signal' promoting inflammation and activating immune cells including granulocytes^13^. CD38 binding CD31 on macrophages negatively regulates TLR4 signalling in those cells, which is particularly relevant as the major *Plasmodium* pathogen‐associated molecular pattern molecules (PAMPs) of glycosylphosphatidylinositol (GPI) and haemozoin (bound to fibrinogen) are known to signal via TLR4 and induce the release of pro‐inflammatory cytokines^14^, ^15^. Taken together, these observations suggest that CD38^+^‐expressing CD4^+^ T cells may play a critical role in immunomodulation during *Plasmodium* infection rather than merely being a marker of 'activated' cells.

Herein, to better understand the role of CD38^+^ CD4^+^ T cells during malaria and in particular during acute malaria, we assessed the genotypic and phenotypic characteristics of the CD38^+^ CD4^+^ T‐cell population present in the peripheral blood of adults presenting to health facilities in Indonesia or Malaysia with acute *P. falciparum* or *P. knowlesi* malaria. Unexpectedly, in most patients, we observed by flow cytometry a prominent population of CD4^+^ T cells co‐expressing high levels of CD4 and CD38 (CD4^hi^CD38^hi^). Assessment of mRNA expression in FACS‐sorted CD4^hi^CD38^hi^ T cells confirmed increased *CD4* gene expression relative to non‐CD4^hi^CD38^hi^ T cells and a prominent signature of genes associated with regulatory T‐cell function including *LAG3, HAVCR2 (TIM3), IL10*, together with other genes that suggest CD4^hi^CD38^hi^ cells serve a specialised immunoinhibitory role during acute malaria.

## Results

### CD4^hi^CD38^hi^ T cells arise during acute malaria

**Figure 1:**
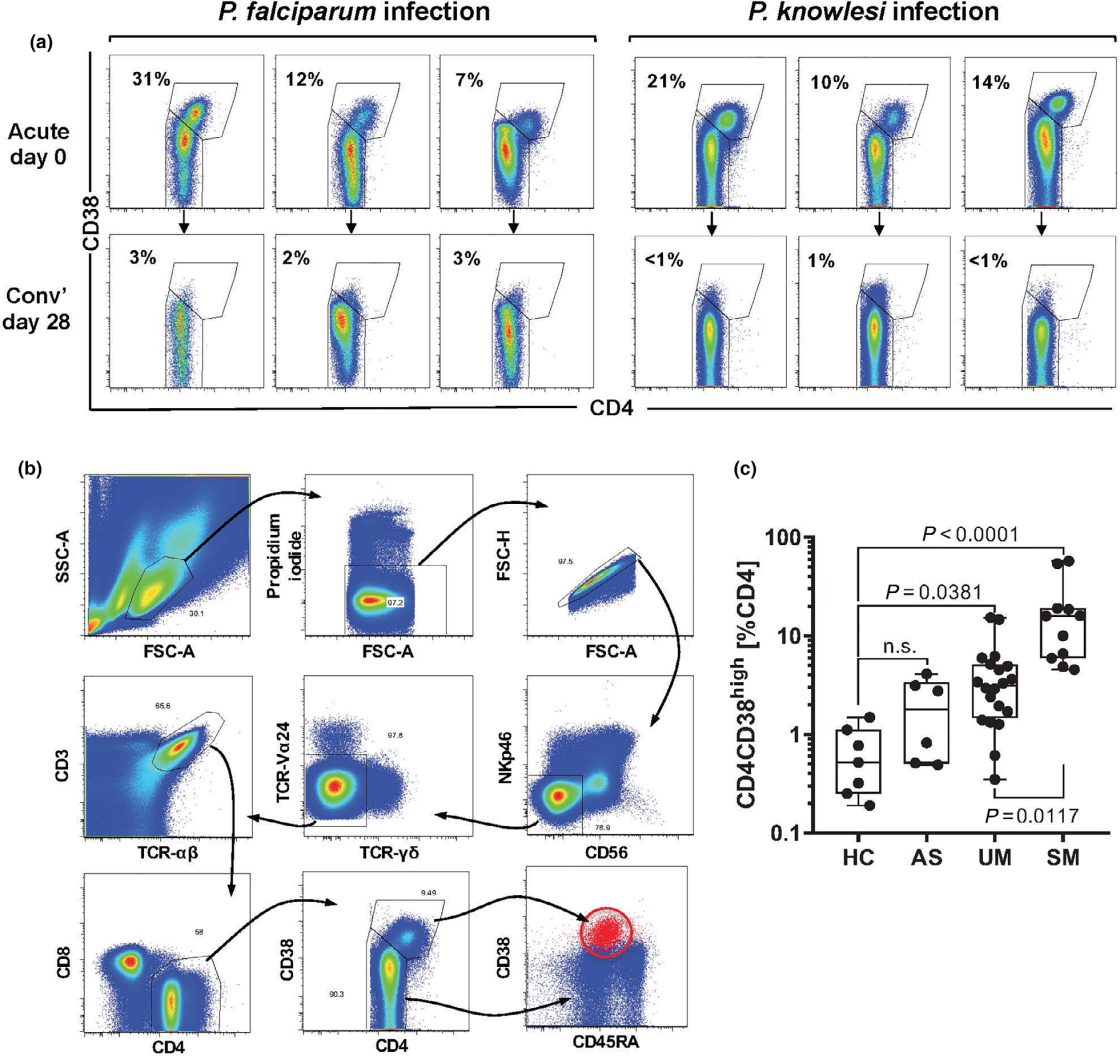
CD4^hi^CD38^hi^ T cells arise during acute malaria. (**a**) Flow cytometric assessment of CD4 and CD38 co‐expression on CD4^+^ T cells in PBMCs of patients with either *P. falciparum* or *P. knowlesi* malaria. Top panels show analysis of samples collected on initial presentation to clinic (acute day 0), and lower panels show matched patient samples collected after drug treatment and convalescence (conv’ day 28). The percentage of CD4^+^ T cells that are CD4^hi^CD38^hi^ are shown. (**b**) Representative gating strategy used to identify CD4^hi^CD38^hi^ T cells. (**c**) PBMCs collected from *Plasmodium*‐negative healthy controls (HC; *n* = 7), *Plasmodium*‐positive, asymptomatic individuals (AS; *n* = 6) and patients with acute uncomplicated *P. falciparum* malaria (UM; *n* = 20) or severe *P. falciparum* malaria (SM; *n* = 11) were analysed by flow cytometry for cell surface marker expression. CD4^hi^CD38^hi^ cells are shown as the percentage of total CD4^+^ T cells. Box plots show the minimum, maximum, median and interquartile range for data from all participants. Horizontal lines depict the median with interquartile range. Data were analysed using the Kruskal–Wallis test followed by post‐tests comparing each group with HC.

We previously identified a significant positive correlation between the frequency of CD4^+^ T cells expressing normal levels of CD4 and increased levels of CD38 (CD4^+^CD38^hi^ cells) and control of parasite growth during the first seven days of primary *Plasmodium* blood‐stage infection in malaria‐naïve volunteers, using a controlled human malaria infection (CHMI) model^8^. However, the function and characteristics of CD38‐expressing CD4^+^ T cells in individuals naturally exposed to malaria, and during an acute malarial infection, are unknown. To determine whether a population of CD4^+^CD38^hi^ cells is present in individuals undergoing secondary exposure to *Plasmodium*, similar to individuals undergoing primary exposure^8^, we performed flow cytometric assessment of CD38 expression on CD4^+^ T cells from PBMCs collected from Malaysian and Indonesian adults who presented to hospitals with acute uncomplicated *P. falciparum* malaria or *P. knowlesi* malaria (before the commencement of drug treatment: acute, day 0) (subject details in Table 1). In most cases of acute falciparum malaria or knowlesi malaria, we observed a distinctive and unexpected population of CD4^+^ T cells that co‐expressed high levels of CD38 and CD4, which we have termed CD4^hi^CD38^hi^ cells (Figure 1a). In paired patient samples taken in convalescence, 28 days following the successful drug treatment, the CD4^hi^CD38^hi^ population was no longer present (Figure 1a). These CD4^hi^CD38^hi^ T cells appear to be an activated subset of conventional peripheral TCR‐αβ CD4^+^ T cells, since they express CD3 and TCR‐αβ, but do not express markers of NK cells (CD56, NKp46), NKT cells (TCR‐Vα24) and γδ T cells (TCR‐γδ) and have lost expression of CD45RA (Figure 1b).

**Table 1:** Study participants (excluding those in Figure 1c)

	P. falciparum malaria (Sabah)	*P. falciparum* convalescent (Sabah d28)	*P. falciparum* malaria (Papua)	*P. knowlesi* malaria (Sabah)
Number, *n*	16	6	6	8
Age, years [median (IQR)]	30 (19–50)	28 (21–50)	28 (26–32)	46 (42–46)
Males, *n* (%)	10 (63%)	6 (100%)	0 (0%)	8 (100%)
Parasitaemia (parasites µL^−1^) [median (IQR)]	9663 (3452–38472)	BDL	10580 (5037–127760)	377 (120–2149)
HRP2 (ng mL^−1^)^a^ [median (IQR)]	126 (28–183)	BDL	588 (318–1020)	NA
Haemoglobin (g dL^−1^) [median (IQR)]	13.2 (11.5–14.6)	13.5 (12.2–14.1)	10.8 (8.2–11.7)	13.5 (12.9–15.2)
Ang‐2 (pg mL^−1^) [median (IQR)]	3086 (1847–7195)	1355 (584–1812)	3554 (2022–6891)	4006 (3231–5841)

All values are median (interquartile range) unless otherwise indicated.

BDL, below detection limit; NA, data not available.

^a^ Samples below detection limit were assigned the value of half the detection limit (0.03 pg mL^−1^).

To investigate the functional relevance of these cells, we evaluated a second cohort of *P. falciparum*‐infected Indonesian adults, to correlate the frequency of CD4^hi^CD38^hi^ cells with disease severity (subject details in Table 2). The frequency of CD4^hi^CD38^hi^ T cells increased with disease severity (Figure 1c). While CD4^hi^CD38^hi^ T‐cell frequency was not significantly increased in asymptomatic (parasitaemic) individuals (AS) compared with local healthy (parasite‐free) controls (HC), it was significantly increased in patients with uncomplicated malaria (UM; *P* = 0.0381), and further increased in patients with severe malaria (SM; *P* = 0.0117) (Figure 1c). Although in parasitaemic patients CD4^hi^CD38^hi^ T‐cell frequency increased with increasing parasitaemia or plasma HRP2, a marker for parasite biomass (both *r* = 0.54; *P* = 0.0006), there was no relationship between CD4^hi^CD38^hi^ T‐cell frequency and either parasitaemia or plasma HRP2, when data were stratified by disease severity (Supplementary figure 1). However, in SM, CD4^hi^CD38^hi^ relative counts (cell frequency) correlated with the endothelial activation marker, Ang2 (*r* = 0.56; *P* = 0.07); this relationship was further supported in a subset with available absolute counts (*n* = 7; *r* = 0.825; *P* = 0.045) (Supplementary figure 2).

**Table 2:** Study participants in Figure 1c

	Healthy controls (HC)	Asymptomatic malaria (AS)	Uncomplicated malaria (UM)	Severe malaria (SM)	*P*‐value^a^
Number, *n*	7	6	20	11	
Age, years [median (IQR)]	30 (25–35)	21 (18–25)	33 (25–41)	25 (23–36)	0.0208
Males, number (%)	6 (86)	6 (100)	13 (65)	7 (64)	
Parasitaemia (parasites µL^−1^) [median (IQR)]	0 (0–0)	85 (62–6956)	2048 (374–7113)	176 758 (91 786–508 775)	< 0.0001
HRP2 (ng mL^−1^)^b^	0.03 (0.03–0.03)	0.3 (0.3–3.1)	34 (0.3–109.6)	5691 (3651–12 247)	0.0003
Haemoglobin (g dL^−1^)	12 (11–16)	11 (9.5–13.25)	11 (10–12.75)	9 (8–12)	0.1142
Angiopoietin‐2 (pg mL^−1^) [median (IQR)]	2511 (1940–3465)	3255 (2666–4698)	3181 (2144–3911)	25 025 (5300–42 177)	< 0.0001

All values are median (interquartile range) unless otherwise indicated.

^a^ Kruskal–Wallis test.

^b^ Samples below the detection limit were assigned the value of half the detection limit (0.03 pg mL^−1^).

### CD4 expression in CD4^hi^CD38^hi^ cells is modulated at the mRNA level

**Figure 2:**
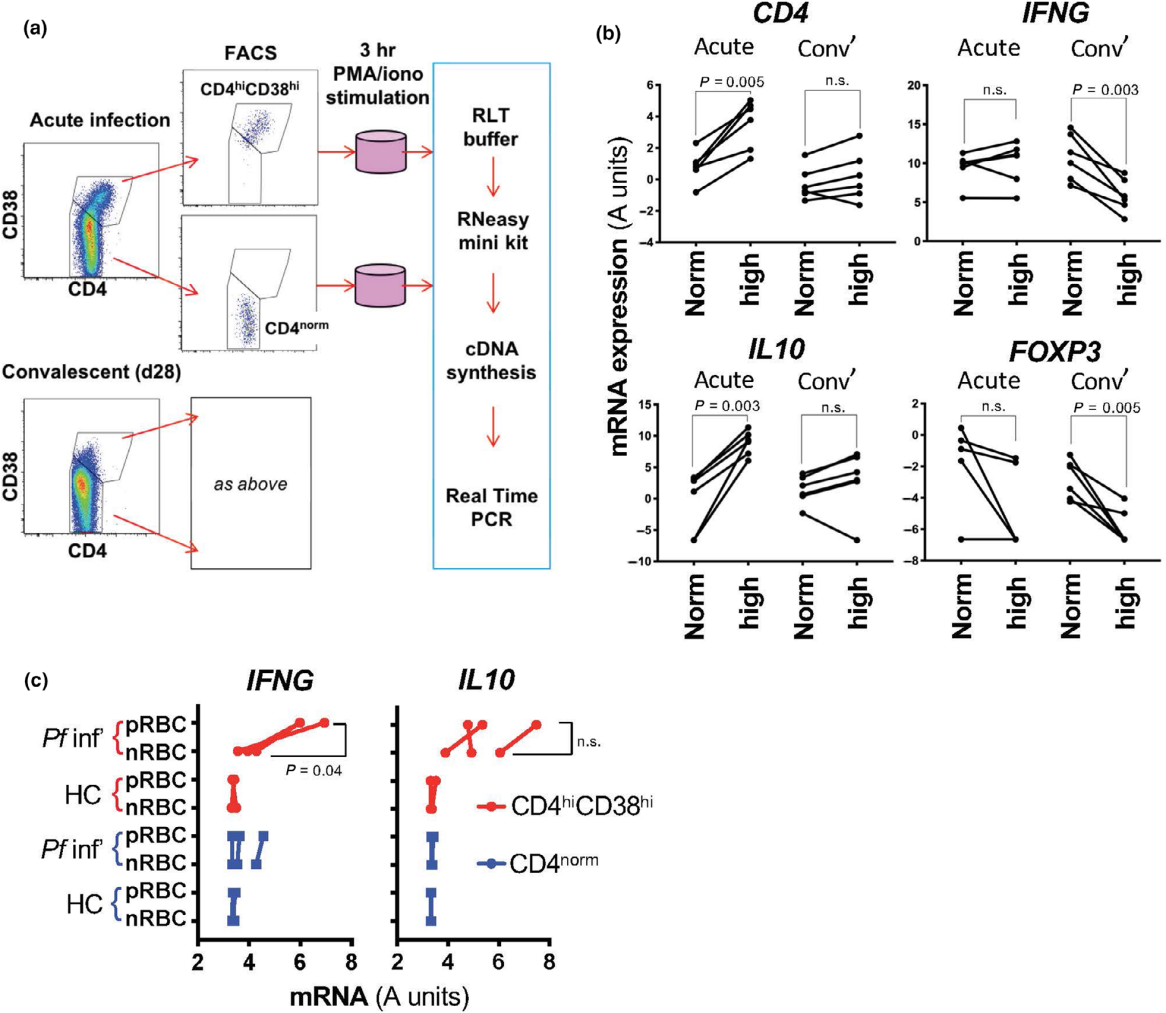
Increased CD4 expression in CD4^hi^CD38^hi^ cells is modulated at the mRNA level. (**a**) Experimental plan for cell sorting, stimulation and gene expression analysis. (**b**) Gene expression assessment by qPCR of FACS‐sorted CD4^norm^ (norm) and CD4^hi^CD38^hi^ (high) T cells from patients with acute *P. falciparum* malaria (*n* = 6, log_2_‐normalised data, paired *t*‐test). (**c**) *P. falciparum* antigen‐specific responses measured in FACS‐sorted CD4^norm^ (blue squares) and CD4^hi^CD38^hi^ (red circles) T cells from patients with acute *P. falciparum* malaria (Pf inf’) or healthy Sabah controls (HC) by qPCR after 24‐h co‐incubation with extract made with *P. falciparum*‐infected RBC (pRBC) or normal RBC (nRBC) (*n* = 3 Pf inf’ and *n* = 3 HC, log_2_‐normalised data, paired *t*‐test).

Upregulation of the CD4 co‐receptor on CD4^+^ T cells has not been described previously in human malaria or in experimental mouse malaria. To confirm that the apparent CD4^high^ population was not an aberration of the flow cytometric analysis, we FACS‐sorted populations of CD4^hi^CD38^hi^ cells and CD4^+^ T cells expressing normal levels of CD4 and intermediate/low levels of CD38 (CD4^norm^) from PBMCs collected from adult patients with acute uncomplicated *P. falciparum* malaria (gating methodology as above, shown in Figure 1b), and then stimulated the cells for 3 h with PMA/ionomycin and assessed mRNA expression in the cells by real‐time qPCR (Figure 2a). Expression of *CD4* mRNA was significantly increased in the CD4^hi^CD38^hi^ population when compared to the CD4^norm^ cells in the acutely infected samples, but not in the convalescent samples (Figure 2b). This suggests that the increased CD4 expression by CD4^high^ T cells is programmed into the cells at the RNA level.

Investigating the relative expression of several other genes, we observed that IFN‐γ mRNA was expressed equally by CD4^hi^CD38^hi^ and CD4^norm^ T cells at the acute stage, but at the convalescent stage the CD4^hi^CD38^hi^ T cells produced significantly less *IFNG* mRNA than the CD4^norm^ cells. This pattern of reduced expression of *IFNG* mRNA in the convalescent samples mirrors our previously published observations for CD4^+^CD38^high^ cells from healthy volunteers (malaria‐naïve Australian volunteers) and for CD4^+^CD38^high^ cells from CHMI volunteers at day 7 of infection^8^. Unlike *IFNG*, CD4^hi^CD38^hi^ cells produced significantly more *IL10* mRNA at the acute stage, but this was lost at the convalescent stage (Figure 2b). Both CD4^hi^CD38^hi^ and CD4^norm^ cells expressed low levels of *FOXP3* (Figure 2b). This pattern of co‐expression of IFN‐γ and IL‐10 together with an absence of FOXP3 expression has been observed in CD4^+^ T cells from children living in malaria‐endemic regions who have suffered recent malarial episodes^16^, ^17^, ^18^ and is associated with a type 1 regulatory T‐cell phenotype (TR1) (reviewed in Roncarolo *et al*.^19^, ^20^). Those studies reported CD4^+^ (CD4^norm^) T cells only and did not identify CD4^hi^ cell populations.

Given the increased frequency of CD4^hi^CD38^hi^ T cells in patients with uncomplicated malaria and severe malaria, compared with asymptomatic but parasitaemic individuals and healthy uninfected controls, we next assessed whether CD4^hi^CD38^hi^ T cells recognise *Plasmodium* antigens. PBMCs collected from adult patients with acute uncomplicated *P. falciparum* malaria were incubated for 24 h with an extract made from *P. falciparum‐*parasitised RBCs (pRBCs) or a control extract made from non‐infected RBCs (nRBCs). Immediately following this *in vitro* stimulation, the CD4^hi^CD38^hi^ and CD4^norm^ cells were sorted by flow cytometry (as above) and placed into RLT lysis buffer for mRNA processing without further stimulation. CD4^hi^CD38^hi^ cells exposed to pRBC responded with an increase in *IFNG* mRNA that was not observed for CD4^norm^ cells or when the control extract was used (Figure 2c). IL‐10 levels were elevated in CD4^hi^CD38^hi^ cells from *P. falciparum* patients regardless of antigen exposure.

### CD4^hi^CD38^hi^ cells express specialised regulatory T‐cell genes

In order to gain a more in‐depth insight into the function and phenotype of CD4^hi^CD38^hi^ T cells, we next utilised the NanoString^TM^ technology platform to assess the differential mRNA expression of 130 genes associated with T‐cell function in CD4^hi^CD38^hi^ and CD4^norm^ cells in acute malaria. CD4^hi^CD38^hi^ and CD4^norm^ cells were sorted from PBMCs collected from acutely infected individuals (Table 1; methodology as above in Figure 2a) with *P. falciparum* infection (6 patients from Papua and 6 from Sabah) or *P. knowlesi* infection (6 patients from Sabah).

**Figure 3:**
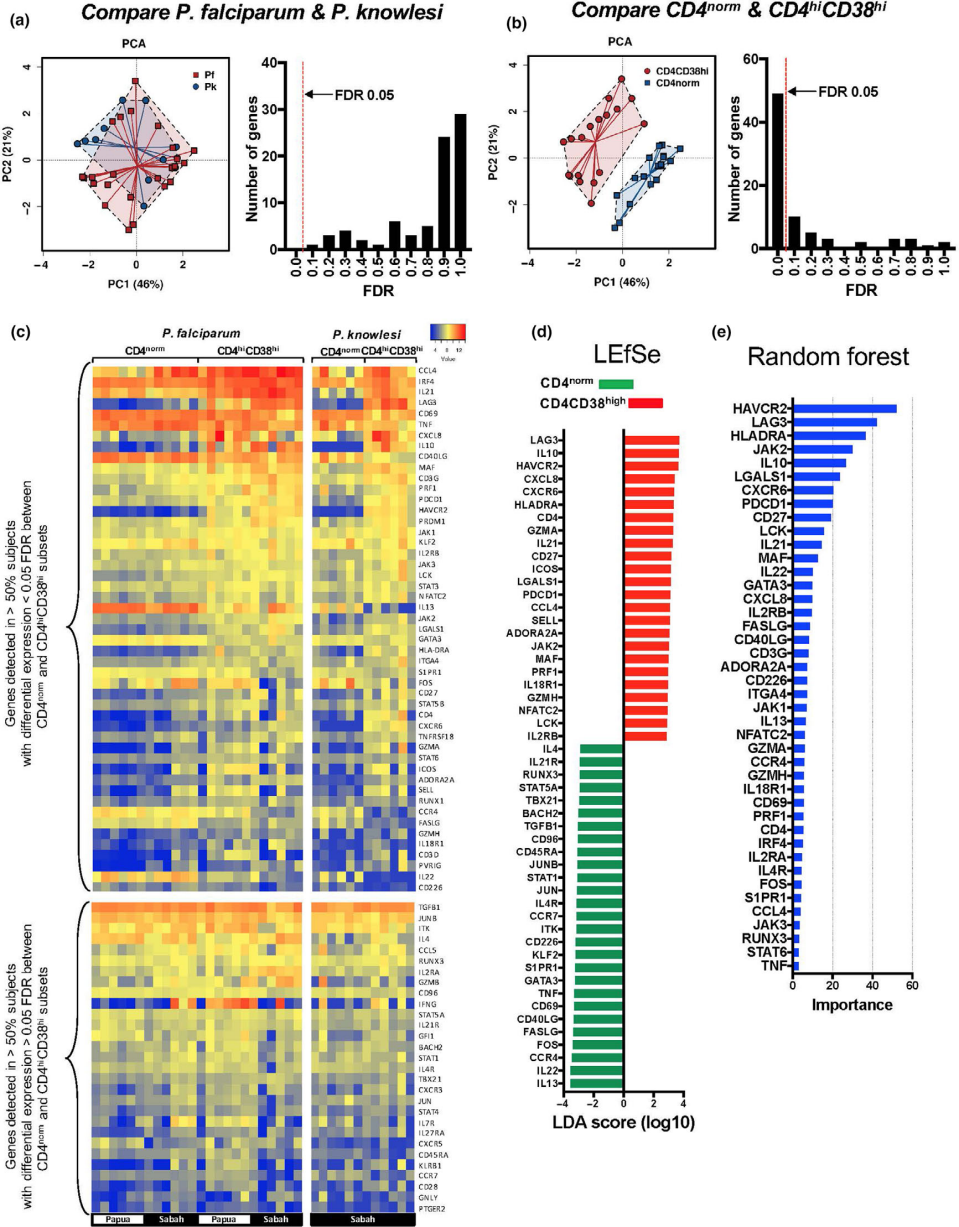
Differential gene expression in CD4^hi^CD38^hi^ T cells. Differential expression of 130 genes associated with T‐cell function was assessed by NanoString in FACS‐sorted CD4^norm^ and CD4^hi^CD38^hi^ T cells from patients attending a clinic with acute *P. falciparum* malaria (Sabah (*n* = 6) and Papua (*n* = 6)) or *P. knowlesi* malaria (Sabah (*n* = 6)). (**a**) Differences in fold changes in gene expression from CD4^norm^ to CD4^hi^CD38^hi^ T cells were analysed by principal component analysis (PCA) comparing cells from patients with *P. falciparum* and *P. knowlesi* malaria (left panel), and histogram of false discovery rate (FDR) was assessed by ANOVA (right panel). (**b**) PCA comparing gene expression in CD4^norm^ and CD4^hi^CD38^hi^ T cells for combined *P. falciparum* and *P. knowlesi* samples and histogram of FDR from a paired *t*‐test (*n* = 18). (**c**) Heatmap of 78 genes reproducibly detected across all samples, showing 49 genes with a FDR < 0.05 in the upper panel (as in 3b above) and the remainder in the lower panel. (**d**) Linear discriminant effect size analysis (LEfSe) using fold changes in gene expression from CD4^norm^ to CD4^hi^CD38^hi^ T cells for all 78 detected genes. (**e**) Random Forest analysis performed on the same data set (truncated at *importance value* 3).

Of the 130 genes, 78 were reproducibly detected across the groups and were included in further analysis (Supplementary table 1). We first assessed differences between the *P. falciparum* (*n* = 12) and *P. knowlesi* (*n* = 6) samples with a principal component analysis (PCA) using the Genomics Data Miner (GMine)^21^, and also by comparing the fold change in gene expression between the CD4^hi^CD38^hi^ and CD4^norm^ cells by ANOVA (Figure 3a). Since our analysis revealed no significant differences between the *P. falciparum* and *P. knowlesi* data sets (FDR < 0.05), we combined those data and performed a PCA comparing differential gene expression between CD4^hi^CD38^hi^ and CD4^norm^ cells, as well as a paired *t*‐test of CD4^hi^CD38^hi^ and CD4^norm^ cells from each subject (*n* = 18) (Figure 3b). In total, 49 genes were significantly differentially expressed between the CD4^hi^CD38^hi^ and CD4^norm^ cells (FDR < 0.05, Supplementary table 1 and Figure 3b and c).

Next, we performed a linear discriminant effect size analysis (LEfSe)^22^ using GMine to identify features most likely to explain the differences between the CD4^hi^CD38^hi^ and CD4^norm^ populations (using all 78 detected genes) (Figure 3d); and a Random Forest analysis to identify the most relevant features of the cohort (Figure 3d) (GMine). Both analyses identified expression of a distinct set of genes associated with T reg function in the CD4^hi^CD38^hi^ cells, including genes strongly associated with TR1 cells^19^: *IL10, LAG3, HAVCR2 (TIM3), PDCD1 (PD1), IL21, ICOS, MAF and PRF1*. Furthermore, another canonical TR1 cytokine, *TGFB1*, was very highly expressed in both the CD4^hi^CD38^hi^ and the CD4^norm^ subsets (but not differentially expressed) (Figure 3c, *lower panel*).

### Insight into the ontogeny of CD4^hi^CD38^hi^ cells

**Figure 4:**
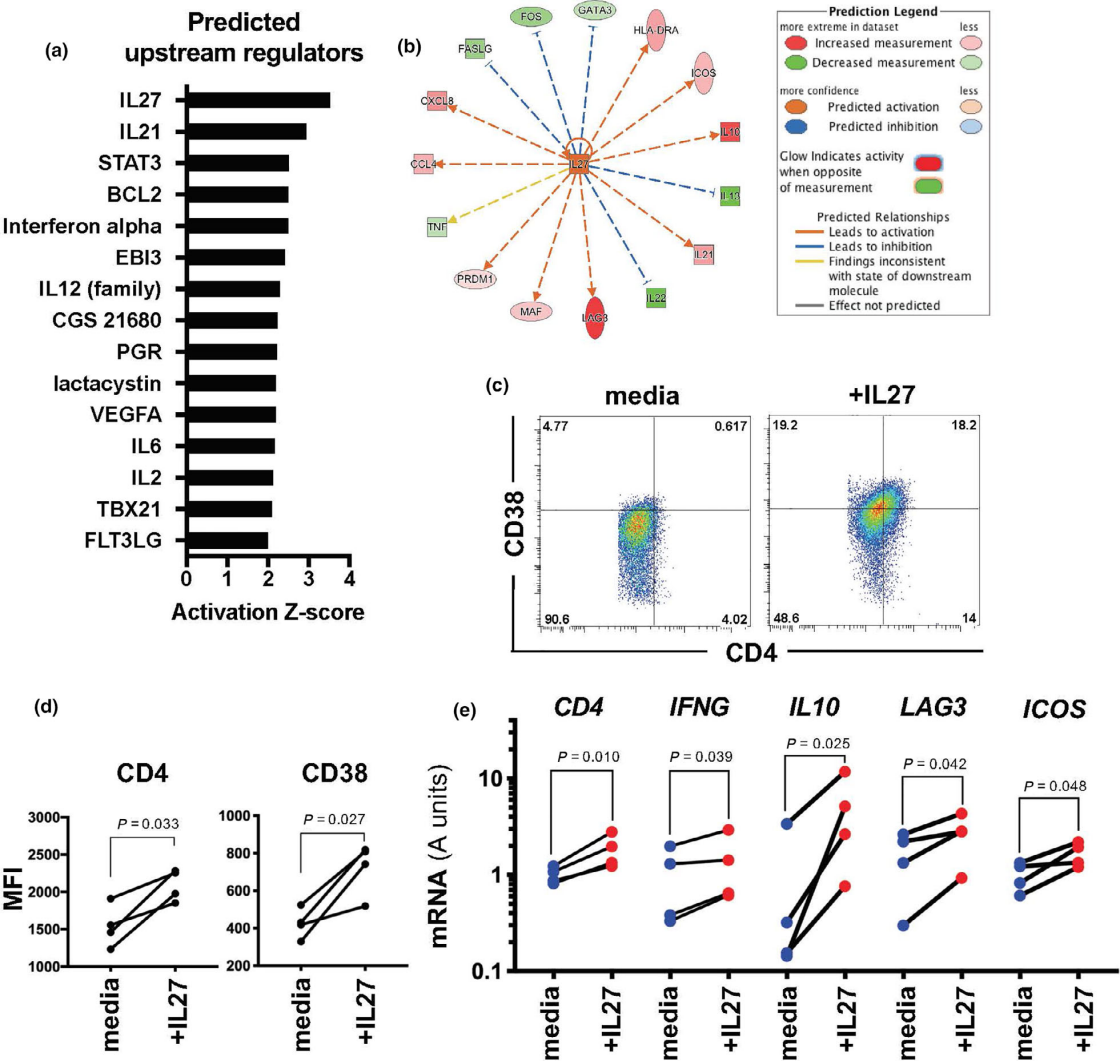
Ontogeny of CD4^hi^CD38^hi^ T cells. (**a**) Predicted upstream regulators of the CD4^hi^CD38^hi^ cell type identified by Ingenuity Pathway Analysis (IPA) of fold changes in gene expression from CD4^norm^ to CD4^hi^CD38^hi^ T cells for the 49 genes identified above with a FDR < 0.05 (truncated at Z‐score = 2); and (**b**) plot of predicted relationships (IPA). (**c**) Representative FACS plots from one volunteer and (**d**) differential expression of CD4 and CD38 on CD4^+^ T cells assessed by FACS in PBMCs from healthy volunteers (Brisbane) cultured for 2 weeks with pRBC extract in media containing IL‐2 (media) or IL‐2 supplemented with IL‐27 (+IL‐27) (*n* = 4, paired *t*‐test). (**e**) Gene expression assessment by qPCR of FACS‐sorted CD4^+^ T cells from PBMCs cultured as above (*n* = 4, log_2_‐normalised data, paired *t*‐test).

We utilised the Ingenuity Pathway Analysis platform and the data generated in the NanoString study (above) to predict upstream regulators of the CD4^hi^CD38^hi^ cells. Using this method, the cytokine IL‐27 was identified as a potential inducer of these cells (Figure 4a and b). To test whether IL‐27 could indeed induce the CD4^hi^CD38^hi^ cell population *in vitro*, we cultured PBMCs from healthy malaria‐naïve (Brisbane) volunteers with *P. falciparum*‐parasitised RBC extract for 14 days +/− recombinant human IL‐27. Cells cultured in the presence of IL‐27 displayed significantly increased co‐expression of both CD4 and CD38 by flow cytometry (Figure 4c and d), and sorted bulk CD4^+^ T‐cell populations from these cultures displayed enhanced *CD4, IFNG, IL10* and *LAG3* mRNA expression (Figure 4e).

### CD4^hi^CD38^hi^ cells in mice

**Figure 5:**
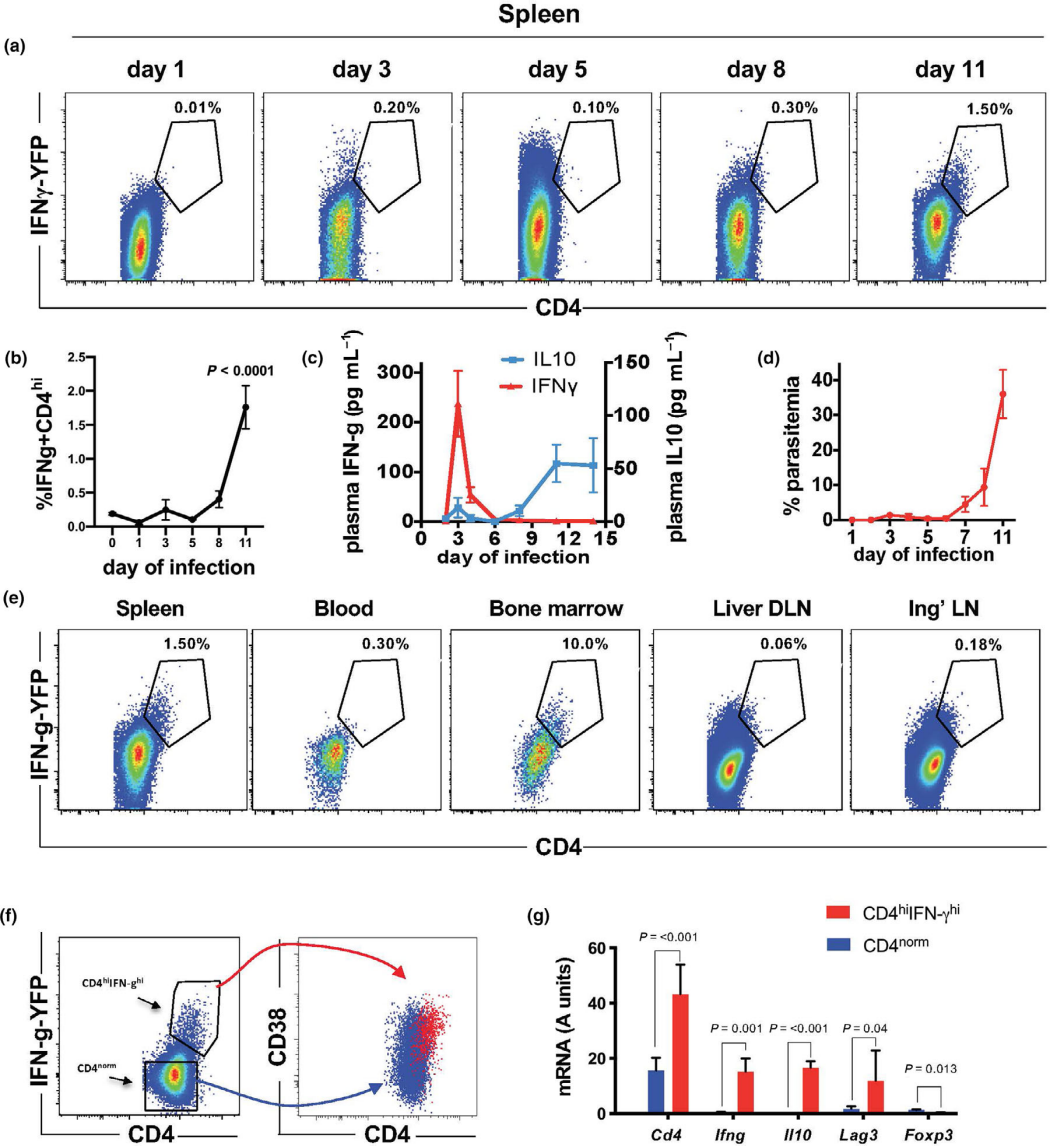
Identification of CD4^hi^ cells in the mouse. (**a**) Kinetic analysis of CD4 and IFN‐γ expression by FACS in CD4^+^ T cells in the spleens of mice (GREAT IFN‐γ reporter mice) infected with *P. yoelii* 17XNL. The percentage of CD4^+^ T cells that are CD4^hi^ and IFN‐γ^hi^ are shown (*n* = 3 mice at each timepoint, concatenated FACS data shown). (**b**) Kinetic analysis of the frequency of CD4^hi^ and IFN‐γ^hi^ in the spleen (*n* = 3 mice at each timepoint, one‐way ANOVA with Dunnett’s multiple comparison test). (**c**) IFN‐γ and IL‐10 levels were assessed by cytokine bead array in the plasma of C57BL/6 mice infected with *P. yoelii* 17XNL at the timepoints indicated. Left axis indicates IFN‐γ and right axis IL‐10 (*n* = 10, mean and SEM shown). (**d**) Parasitaemia in GREAT IFN‐γ reporter mice following the infection with *P. yoelii* 17XNL (*n* = 3, mean and SEM shown). (**e**) Analysis of CD4 and IFN‐γ expression by FACS on day 11 of infection with *P. yoelii* 17XNL in CD4^+^ T cells in the organs indicated in GREAT IFN‐ reporter mice. The percentage of CD4^+^ T cells that are CD4 and IFN‐γ positive are shown (DLN = draining lymph node, Ing LN = inguinal lymph node). (**f**) FACS analysis of CD38 expression in CD4^hi^IFN‐γ^hi^ cells in GREAT IFN‐γ reporter mice on day 11 of *P. yoelii* 17XNL infection. (**g**) CD4^hi^IFN‐γ^hi^ cells (gated as in **e** above) were FACS‐sorted from the spleens of GREAT IFN‐γ reporter mice on day 11 of infection with *P. yoelii* 17XNL, and gene expression was assessed by qPCR (*n* = 3, log_2_‐normalised data, paired *t*‐test, mean with SD shown). Data shown are from three independent experiments.

We were curious as to why no analogue of the CD4^hi^CD38^hi^ cell population has been yet reported in mice, given the very large number of immunological studies performed utilising rodent malaria models including those focused on the role of CD4^+^ T cells. Indeed, in our laboratory, despite many thousands of flow cytometry analyses conducted using a flow cytometric method for monitoring *Plasmodium* infection^23^, we have never observed CD4^high^ cells in the blood of mice during infection. To provide a foundation for dissecting the role of CD4^hi^CD38^hi^ cells in host–pathogen immunity, we revisited our *P. yoelii* murine malaria model with a different approach. To identify CD4^+^ T cells specifically responding to infection, we utilised a mouse model where cells are engineered to express the fluorescent protein YFP upon the production of IFN‐γ^24^. Using this model, mice were infected with non‐lethal *P. yoelii* 17XNL‐parasitised RBC via intravenous injection to establish a blood‐stage infection. On days 1, 3, 5, 8 and 11 of infection, cells were recovered from several organs including the spleen, blood, bone marrow, liver‐draining lymph nodes (DLNs) and inguinal lymph nodes (Ing LNs). Using this more‐focused approach, IFN‐γ‐producing CD4^+^ T cells were evident in the spleen at day 3 of infection (Figure 5a), but by day 8, the YFP^+^ CD4^+^ T cells were diminished, and by day 11, the remaining YFP^+^ CD4^+^ cells in the spleen were also CD4^high^ (Figure 5a and b). This pattern of IFN‐γ expression coincides with a peak in plasma IFN‐γ routinely observed in mouse models^25^, ^26^, ^27^, and in our own experiment in C57BL/6 mice (Figure 5c) in which plasma IFN‐γ peaked around day 3 and IL‐10 around day 11. This timing coincides with the onset of acute parasitaemia, which can reach very high levels in the *P. yoelii* mouse model (Figure 5d). These CD4^high^ cells were apparent only in the spleen and bone marrow, and not in blood, liver DLN or inguinal LN (Figure 5e). The CD4^high^ cells from the spleen at day 11 were also CD38^high^, but the levels of CD38 were not high enough to make this population as prominent as observed in the human studies (Figure 5f). Finally, to assess gene expression in the CD4^high^ murine cells, CD4^high^ and CD4^norm^ cells were FACS‐sorted from spleens collected at day 11 of infection and processed directly for analysis by real‐time qPCR without further stimulation. As in human CD4^hi^CD38^hi^ cells, murine CD4^hi^ cells had increased *CD4* mRNA and contained within this population were the *IL10*‐producing cells with increased *LAG3* and an absence of *FOXP3* (Figure 5g). Thus, the population identified in the *P. yoelii* murine model shares core similarities with the CD4^hi^CD38^hi^ population in humans.

## Discussion

We have identified a population of CD4^+^ T cells in the blood of acute malaria patients infected with *P. falciparum* or *P. knowlesi* that co‐express high levels of the CD4 co‐receptor and the ectoenzyme CD38. These CD4^hi^CD38^hi^ T cells appear to be an activated subset of conventional peripheral TCR‐αβ CD4^+^ T cells, since they express CD3 and TCR‐αβ but do not express markers of NK cells (CD56, NKp46), NKT cells (TCR‐Vα24) or γδ T cells (TCR‐γδ) and have lost expression of CD45RA. Because of reductions in absolute CD4 T‐cell numbers in the blood during acute malaria, NK cells are relatively more abundant^28^; hence, we excluded these based on the expression of CD56. These CD4^hi^CD38^hi^ cells also expressed a set of genes strongly associated with immunomodulatory function, including *IL10*, *LAG3* and *TIM3* (*HAVCR2*), and were present in the peripheral blood of patients in malaria‐endemic areas during acute symptomatic infection but were absent following the drug treatment and convalescence.

CD4^+^ T cells play a critical role in the control of the blood stage of *Plasmodium* infection, both in humans and in animal models (reviewed in Kurup *et al*.^4^, ^29^); however, a clear role also exists for CD4^+^ T cells in suppressing immunopathology. The appearance of IL‐10‐expressing CD4^+^ T cells during *Plasmodium* infection in humans^16^, ^17^, ^18^, ^30^, ^31^ and mice^32^, ^33^ has been well documented, and early reports associated cerebral malaria in children with high circulating IL‐10 levels^34^, ^35^, ^36^: one study found IFN‐γ/IL‐10‐co‐producing cells in children with severe malaria^17^.

CD4^hi^CD38^hi^ cells appear to provide a more comprehensive set of markers for TR1 regulatory T cells as they strongly express canonical TR1 genes including *IL10*, *IFNG*, *TGFB1*, *LAG3*, *TIM3*, *IL21*, *ICOS*, *PD1* (*PDCD1*), *MAF*, and *PRF1* and do not express *FOXP3* (reviewed in Roncarolo *et al*.^19^, ^20^). CD4^hi^CD38^hi^ cells strongly express *CXCL8* (IL‐8), which is a pro‐inflammatory cytokine not previously associated with TR1 cells but shown to be produced by human FOXP3^+^ T regs^37^ and upregulated by CD4^+^ T cells very early during primary *Plasmodium* infection^38^. Our cell population is also distinct from the CD38‐expressing CD4^+^ T‐cell subset identified previously as a hypoproliferative IL‐13‐secreting CD4^+^ T‐cell subset that retained expression of naïve cell surface markers following the TCR stimulation and secreted IL‐13, and IFN‐γ and TNF^39^. Note that the CD4^hi^CD38^hi^ population described in our study may or may not be a homogeneous population.

Our pathway analysis (Figure 4) suggested IL‐27 as an upstream regulator of the CD4^hi^CD38^hi^ phenotype. *In vitro* studies with mice have identified IL‐27 as an inducer of IL‐10 production in CD4^+^ T cells^40^, ^41^ and confirmed a role for IL‐27 receptor signalling^41^, and similar observations were made with *in vitro* cultures of human cells^42^. In the context of experimental rodent malaria, IL‐27 (acting via the IL‐27 receptor) was shown to induce the generation of IL‐10‐expressing CD4^+^ T cells that were critical to limit immunopathology^32^, ^43^. It remains possible that other cytokines may also induce the CD4^hi^CD38^hi^ phenotype; indeed, our pathway analysis (Figure 4) also identified IL‐12 and IL‐21 as potential upstream regulators and others have shown that IL‐6 and TGF‐β could induce IL‐10 production by CD4^+^ T cells in mice^41^. The mechanisms of CD4^hi^CD38^hi^ cell generation *in vivo* remain to be elucidated, but we think it likely that IL‐27 levels *in vivo* are likely to be higher around areas of antigen presentation and more likely to affect cells responding to antigen. This may explain the fairly uniform upregulation of CD4 and CD38 we observed in our *in vitro* cultures. Possibly adding support to the notion that IL‐27 is driving the CD4^hi^CD38^hi^ phenotype in our patients with acute malaria is a recent paper by Oterdal *et al*. who found that IL‐27 levels are elevated in the plasma of adults living in malaria‐endemic areas with acute falciparum malaria^44^.

A striking feature of the CD4^hi^CD38^hi^ cell is its very high expression of the CD4 co‐receptor. The canonical role of the CD4 co‐receptor is to bind a region on the MHC‐II molecule and restrict signalling through the CD4^+^ T‐cell receptor to antigens bound only on MHC‐II molecules^45^. However, the CD4 co‐receptor serves multiple other roles including acting as a receptor for the chemotactic cytokine IL‐16^46^, and facilitating adhesion to cells expressing MHC‐II independently of TCR engagement (reviewed in Glatzova *et al*.^47^). The latter is particularly relevant in malaria as MHC‐II expression is dramatically increased on murine brain microvascular endothelium during experimental cerebral malaria^48^, and on human endothelium in response to IFN‐γ^49^.

Another vital role of the CD4 co‐receptor is to enhance signalling through the T‐cell receptor during engagement with the peptide–MHC‐II complex^50^. An analogous effect has been observed in CD8^+^ T cells, by us^51^ and others^52^, where the sensitivity of the T‐cell receptor was shown to be modulated by increasing or decreasing the expression levels of the CD8 co‐receptor. This can increase the range of specificity of the T cell, thereby allowing a T cell that is typically restricted to a very specific peptide to be able to respond in a cognate fashion to a broader range of targets. In the study reported herein, CD4^hi^CD38^hi^ cells responded to parasitised RBC by strongly expressing IFN‐γ and IL‐10, but did not respond to uninfected RBC controls, suggesting that TCR signalling is functioning in these cells and that they are not exhausted^53^. However, we cannot rule out the possibility that some cells responding to antigen may have started out as CD4^norm^ prior to the *in vitro* assay.

Downregulation of the CD4 co‐receptor has been reported in one study in mice^54^, and several other studies have demonstrated increased CD4 expression on CD4^+^ T cells following the very strong or chronic activation, such as in a mixed lymphocyte reaction^55^ or an alloreactive transplant setting^56^, ^57^. However, increased CD4 expression in the context of natural host–pathogen interactions, as shown herein for our novel CD4^hi^CD38^hi^ cell population, has not been previously reported. Importantly, we show that this increase in CD4 expression is modulated at the mRNA level, providing the first evidence of this fundamental insight into T‐cell biology. This is significant because CD4 levels could be transiently modulated by cycling of the co‐receptor between the cell surface and cytoplasm^58^; however, increased *CD4* gene expression together with the required energy expenditure emphasises the level of commitment to the cell phenotype, suggesting that increased CD4 expression is important to the function of these cells.

The unusual characteristics of CD4^hi^CD38^hi^ cells are well suited to a specialised T regulatory cell arising in response to *Plasmodium* infection. Firstly, in residents of two distinct malaria‐endemic areas (Sabah and Papua), CD4^hi^CD38^hi^ cells were present in relatively large numbers during acute malaria but were absent in convalescence following the successful drug clearance of parasites, and CD4^hi^CD38^hi^ cells could recognise *Plasmodium*‐infected RBC *in vitro*, suggesting that CD4^hi^CD38^hi^ cells are responding directly to *Plasmodium* infection. Secondly, CD4^hi^CD38^hi^ cells express a unique set of genes with particular relevance to *Plasmodium* infection, including those mentioned above and *CXCR6* whose ligand (CXCL16) is constitutively expressed in cerebrospinal fluid^59^, on endothelial cells in the brain^60^ and in the placenta during malaria^61^, and may induce migration and localisation of CD4^hi^CD38^hi^ to these tissues. CD4 would promote localisation to areas of increased MHC‐II expression during infection, and CD38 is a ligand for CD31 (platelet endothelial cell adhesion molecule, PECAM‐1) whose expression on endothelial cells is upregulated during malaria^62^, which may in turn promote CD4^hi^CD38^hi^ cell localisation. Consistent with this, it was notable that the frequency of CD4^hi^CD38^hi^ cells in severe malaria was strongly associated with measures of endothelial activation. CD38 is also a critical ectoenzyme catalysing extracellular nicotinamide adenine dinucleotide (NAD), thereby regulating extracellular levels of NAD released by lysis of infected RBC^12^, reducing levels of pro‐inflammatory NAD^13^ and providing the substrate ADPR that is further catalysed to generate the strongly immune‐inhibiting molecule adenosine^63^. Another role for CD38 is to bind CD31 on macrophages and inhibit TLR4 signalling, which is relevant since the major *Plasmodium* PAMPs of glycosylphosphatidylinositol (GPI) and haemozoin (bound to fibrinogen) are known to signal via TLR4 and induce the release of pro‐inflammatory cytokines^14^, ^15^.

Taken together, our data suggest that CD4^hi^CD38^hi^ cells are a specialised TR1 cell that arise during acute infection. We propose a role in limiting immunopathology. In our study, CD4^hi^CD38^hi^ cells were increased in proportion to malaria disease severity (Figure 1c), and there are phenotypic analogies between the CD4^hi^CD38^hi^ T cells identified herein and IL‐10‐expressing CD4^+^ T cells that were shown to limit immunopathology in a rodent malaria model^32^. Whether these cells also inhibit parasite clearance is not known. Studies in rodents have shown that blocking T regulatory cell function can enhance parasite clearance^33^, ^64^, and Jagannathan *et al*. found that the CD4^+^ T cells co‐expressing IFN‐γ and IL‐10 may associate with an increased risk of future malaria in children^17^. In a previous study, we found that control of the parasite following the infection of malaria‐naïve volunteers with *P. falciparum*‐infected RBC was associated with an increased frequency of CD4^+^ T cells co‐expressing CD38, but these did not have increased expression of CD4, were poor producers of IFN‐γ^8^ and appeared to be analogous to mouse CD4 T cells activated *in vitro* ^65^ and markedly different from those we describe herein during clinical disease. Whether the CD38‐expressing CD4^+^ T cells associated with treatment failure in clinical malaria in Uganda had increased expression of CD4 was not reported. While there was no relationship between CD4^hi^CD38^hi^ cells and parasite biomass evident in the current study, parasite clearance could not be assessed, and the role of this cell population in parasite clearance requires prospective study.

In conclusion, we describe herein a population of CD4^+^ T cells expressing high levels of CD4 and CD38 (CD4^hi^CD38^hi^ cells) in adult patients acutely infected with either *P. falciparum* or *P. knowlesi*. Assessment of differential mRNA expression of 130 genes associated with T‐cell function revealed that these CD4^hi^CD38^hi^ cells had a TR1 phenotype, with high expression of *LAG3*, *IL10*, *TIM3* and numerous other genes associated with modulatory activity. We further provide the first naturally occurring example of increased CD4 expression on CD4^+^ T cells, and the first evidence that changes in CD4 co‐receptor expression levels are modulated at the mRNA level. These findings give new insight into T‐cell biology and provide a starting point for future studies that aim to enhance *Plasmodium* parasite clearance while avoiding immunopathology.

## METHODS

### Study participants

In Sabah, an area of low malaria transmission, patients were enrolled from a tertiary referral centre (Queen Elizabeth Hospital 1, Kota Kinabalu)^66^, ^67^ and two district hospitals (Kudat and Kota Marudu) as part of concurrent prospective clinical studies^68^. During the sample collection period, the region was co‐endemic for *P falciparum*, *P. vivax* and the zoonotic parasite *P. knowlesi* ^66^, ^69^. Criteria for enrolment included a blood film that was positive by microscopy for any *Plasmodium* species, fever or history of fever in the last 48 h, no major concurrent illness or comorbidity, and no prior antimalarial therapy in the preceding 24 h. Patients were excluded if pregnant or lactating. Hospitalisation was mandatory in all patients with malaria in Sabah. All patients with malaria were treated according to the local guidelines. Uninfected participants were visitors or relatives of patients with malaria, with no fever or history of fever in the preceding 14 days and with a blood film negative for malaria parasites. Heparinised venous blood samples were collected at presentation, and follow‐up samples were collected 28 days post‐treatment. Peripheral blood mononuclear cells (PBMCs) were isolated and cryopreserved until analysis.

In a lowland region of Papua with perennial, unstable transmission of *P. falciparum*, *P. vivax* and *P. malariae,* patients were enrolled with malaria attending the Mitra Masyarakat Hospital in Timika or a regional health clinic. The following groups of participants were enrolled following the informed consent, and patients with acute uncomplicated falciparum malaria (UM) presenting with *P. falciparum* monoinfection by microscopy, fever or history of fever in the preceding 48 h and no alternative cause were identified (*n* = 20)^70^, ^71^, ^72^: patients with *P. falciparum* parasitaemia and ≥ 1 modified WHO criteria of severe malaria (SM), acute renal failure (creatinine > 265 µmol L^−1^), or hyperbilirubinaemia with renal impairment (creatinine > 130 µmol L^−1^) and/or parasitaemia of > 100 000 parasites μL^−1^, or blackwater fever, or hyperparasitaemia (> 10% parasitised red cells), or cerebral malaria (Glasgow coma score < 11), or hypoglycaemia^72^. In the SM cohort (*n* = 11), 3 patients (27%) had cerebral malaria, 3 patients (27%) had hyperparasitaemia, 5 patients (45%) had renal failure, 4 patients (36%) had hyperbilirubinaemia, and 7 patients (64%) had more than one WHO criterion for severe disease. Asymptomatic malaria‐exposed adults, resident in Timika district for at least two years, with no fever or symptoms of malaria within the preceding two weeks were enrolled as controls. Those without parasitaemia were grouped as healthy controls (*n* = 7) and those with asymptomatic parasitaemia as asymptomatic controls (*n* = 6). Peripheral blood mononuclear cells (PBMCs) were isolated from heparinised venous blood samples and cryopreserved until analysis.

### Study approval

The human studies were approved by the ethics committees of the Malaysian Ministry of Health, the Indonesian National Institute of Health Research and Development, the NT Department of Health and Menzies School of Health Research and QIMR Berghofer Medical Research Institute. Written informed consent was obtained from all participants.

### Plasma biomarkers

Total body parasite biomass was quantified by detecting plasma concentration of *P. falciparum* Histidine Rich Protein 2 (PfHRP2) using ELISA as described^73^. Plasma Ang‐2 was measured as a biomarker of endothelial activation by ELISA (Quantikine, R&D Systems) as previously described^74^.

### Flow cytometry

PBMCs isolated from adult patients with *P. falciparum* or *P. knowlesi* malaria in Papua or Sabah were cryopreserved and kept in liquid N2. Frozen samples were thawed in 10 volumes of complete media (RPMI 1640 containing phenol red supplemented with 10% FCS and 50 mg L^−1^ gentamicin; Sigma‐Aldrich). All antibodies were titrated before experimental use with PBMCs from healthy volunteers to determine the optimal staining concentration. Cells were stained with 50 μL of FACS staining buffer (PBS supplemented with 0.5% FCS and 2 mM EDTA) containing combinations of anti‐human antibodies as detailed in the text including the following: CD3‐AF700 (BioLegend, clone OKT3), CD4‐BV510 (BioLegend, clone OKT4), CD8‐APC‐H7 (BD Biosciences, clone SK1), CD38‐PerCP/Cyanine5.5 (BioLegend, clone HB‐7), TCR‐α/β‐PE/Cy5 (BioLegend, clone IP26), CD56‐PE‐Cy7 (BD Biosciences, clone B159), TCR‐γ/δ‐AF‐647 (BioLegend, clone B1), TCR‐Vα24‐Jα18‐FITC (BioLegend, clone 6B11), CD335‐BV421 (BioLegend, clone 9E2), CD45RA‐BV‐606 (BioLegend, clone HI100) and 1 μL of human Fc receptor blocking solution (Human TruStain FcX, BioLegend) for 30 min at room temperature. Cells were washed and resuspended in FACS staining buffer containing propidium iodide (Thermo Fisher Scientific) before acquisition or sorting. In some experiments (e.g. data shown in Figure 1c), 400 000 PBMCs in PBS/2% FCS were stained with anti‐CD3‐FITC (HIT3a), CD4‐PerCP (RPA‐T4) and CD38‐APC (HIT2) (all from BD Biosciences) for 30 min on ice. Cells were washed twice and fixed with 1% paraformaldehyde (Sigma, Australia) before acquisition.

For flow cytometry experiments with mice, single‐cell suspensions from organs as detailed in the text were prepared and stained with combinations of anti‐mouse antibodies including the following: CD3‐PE/Cy5 (BioLegend, clone 145‐2C11), CD3‐V450 (BD Biosciences, clone 500A2), CD4‐PE (BioLegend, clone GK1.5), CD4BV510 (BioLegend, clone RM4‐5), GR‐1‐PE‐Cy7 (BD Biosciences, clone RB6‐8C5), CD11a‐AF647 (BioLegend, clone M17/4), CD19‐AF700 (BioLegend, clone 6D5), CD8‐APC‐Cy7 (BioLegend, clone 53‐6.7), CD11b‐BV711 (BioLegend, clone M1/70), CD11c‐BV785 (BioLegend, clone N418) and CD38‐PerCp/Cyanine5.5 (BioLegend, clone 90). Propidium iodide or Sytox Red (Thermo Fisher Scientific) was added to the running buffer. Data acquisition was performed on a LSRFortessa 4 instrument (BD Biosciences) using Diva software. Cell sorting was performed using a BD Aria III cell sorter (BD Biosciences). FlowJo software version 9.9 was used for post‐acquisition gating and analysis.

For data shown in Figure 1c, 400 000 PBMCs in PBC/2% FCS were stained with anti‐CD3‐FITC (HIT3a), CD4‐PerCP(RPA‐T4) and CD38‐APC (HIT2) (all from BD Biosciences) for 30 min on ice. Cells were washed twice and fixed with 1% paraformaldehyde (Sigma, Australia), and samples were acquired on a FACSCalibur (BD Biosciences) using the CellQuest software. Data were analysed using the Kaluza software (Beckman Coulter).

### Gene expression analysis by qPCR

Cells as detailed in the text were sorted by FACS directly into RLT buffer and stored at −80°C. On the day of extraction, frozen cell lysates were thawed quickly on ice and mRNA was extracted using the RNeasy Micro Kit (Qiagen) according to the manufacturer's instructions. cDNA was synthesised using oligo‐dT and Superscript III (Invitrogen) according to the manufacturer's instructions. Gene expression was measured using individual TaqMan gene expression assays (Applied Biosystems) and Platinum Taq Polymerase (Life Technologies) in a 15 μL volume reaction using a Rotor‐Gene 3000 PCR machine (Corbett Research) with the following conditions: 2 min at 50°C for calibration of fluorescent gain values, then denaturing for 2 min at 95°C, followed by 45 cycles of 5 s at 95°C and 30 s at 60°C. Gene expression was quantified relative to a standard curve generated from a titration of cloned cDNA, and relative gene expression was calculated by dividing the expression value of the test gene by that of a reference gene (RPL13A for human studies and β2m for mouse study) except for the data in Figure 4d, which was normalised as molecules/cell as the input cell number was accurately determined beforehand.

### T‐cell stimulation with *Plasmodium* antigens

PBMCs (4 × 10^5^) from adult patients with uncomplicated *P. falciparum* malaria (Sabah) were co‐cultured for 24 h in 96‐well U‐bottom plates with RPMI supplemented with 10% human AB serum and with an extract equivalent to 4 × 10^5^ *P. falciparum‐*parasitised RBCs (pRBCs), or a control extract made from equivalent numbers of uninfected RBCs (nRBCs). The pRBC and nRBC extracts were made as described previously^75^. Following the incubation period, the cells were harvested and sorted by flow cytometry into populations of CD4^hi^CD38^hi^ or CD4^norm^ T cells. Following the FACS sort, the cells were resuspended in RLT lysis buffer and PCR was performed as described above.

### mRNA expression using NanoString

Expression of 130 immune‐related genes known to be involved in T‐cell and B‐cell responses was assessed using a custom 130‐plex nCounter codeset of genes involved in immune recognition, survival, migration, adhesion, cytokine/chemokine secretion, activation, differentiation and exhaustion, as reported previously^76^. CD4^norm^ and CD4^hi^CD38^hi^ T cells were sorted by FACS from PBMCs from samples as detailed in the text. The sorted cells were stimulated for 3 h with PMA (5 ng mL^−1^) and ionomycin (500 ng mL^−1^) in RPMI supplemented with 10% human AB serum and penicillin–streptomycin–glutamine (PSG). Following the stimulation, the cells were washed and resuspended in RLT lysis buffer (2 × 10^3^ cells μL^−1^) and stored at −70°C until use. nCounter codeset hybridisation was performed as per the manufacturer’s instructions using 5 μL of cell lysate (10^4^ cells). Samples were processed using the NanoString GEN2 Prep Station and data acquired using the nCounter Digital Analyzer (NanoString Technologies). Data were normalised to the mean RPLP0 expression levels and processed using nSolver (NanoString Technologies) using the recommended settings. The limit of detection was set as the average reading for the negative controls plus 2 SD; all values below this were raised to this value, and all data were then log_2_‐normalised for statistical analysis. Genes were considered as reproducibly detected if they were above the limit of detection in at least 50% of the samples. Principal component analysis, ANOVA, paired *t*‐tests, LEfSe and Random Forest analysis were performed using the Genomics Data Miner^21^. Heatmaps were generated using Heatmapper^77^, and data were ordered according to average fold change between CD4^norm^ and CD4^hi^CD38^hi^ groups.

### PBMC culture with IL‐27

PBMCs from healthy Brisbane volunteers were co‐cultured with an extract made from *P. falciparum*‐infected RBC (as detailed above) at the equivalent of 1xpRBC:1xPBMC in media alone (RPMI, with 5% human AB serum, PSG and 20 units per mL rIL‐2), or further supplemented with IL‐27 (100 ng mL^−1^) in 2.5 mL at 2.5 × 10^6^ PBMCs/well in 12‐well tissue culture plates for 8 days; then, the cells were split into 2 wells of 6‐well plates with 5 mL of fresh media and supplements and cultured for further 6 days (recombinant IL‐2 was obtained through the AIDS Research and Reference Reagent Program, Division of AIDS, National Institute of Allergy and Infectious Diseases and National Institutes of Health: human rIL‐2 from Dr Maurice Gately, Hoffmann‐LaRoche (Nutley, NJ); recombinant IL‐27 from BioLegend. After 13 days of culture, the cells were restimulated for 24 h with the addition of pRBC extract (equivalent of 10^6^ pRBC mL^–1^); then, the cells were harvested, and 10 × 10^3^ CD4^+^ T cells were sorted by FACS and immediately resuspended into 200 μL of RLT lysis buffer and stored at −80°C until processed for qPCR.

### Mice

Specific pathogen‐free BALB/c and C57BL/6 mice were purchased from the Animal Resources Centre, Perth, WA, and were used at 6 or 9 weeks of age. Interferon‐gamma reporter mice were purchased from the Jackson Laboratories (C.129S4(B6)‐*Ifng^tm3.1Lky^*/J; stock No. 017580) and bred at QIMR Berghofer Medical Research Institute. All studies were approved by the QIMR Berghofer Medical Research Institute Animal Research Ethics Committee.

### Mouse *Plasmodium* infections

To generate stocks of parasitised RBC (pRBC), BALB/c mice were infected by tail‐vein injection of 1000 cryopreserved infectious *P. yoelii* sporozoites (17XNL non‐lethal strain) kindly provided by Dr SL Hoffman (Sanaria Inc., Rockville, MD, USA) and blood‐stage parasitaemia was monitored by FCAB assay^23^. On day 14, the mice were euthanised and the parasitised blood was collected and stored in freezing buffer in liquid nitogen. Blood‐stage infections reported herein were initiated by tail‐vein injection of 10^5^ pRBC, and the infection was monitored daily by FCAB assay^23^. For kinetic analysis of CD4 and IFN‐γ expression in GREAT IFN‐γ reporter mice, GREAT mice were infected with *P. yoelii* as described above, and on the days indicated in the text, three mice were selected randomly for euthanisation, and organs and blood were collected for analysis. Single‐cell suspensions were prepared and analysed by flow cytometry. Cytokine measurement in mouse plasma was assessed by cytokine bead array (BD Biosciences) using 10 μL of plasma taken from the tail tip of infected mice on the days shown in the text.

### Statistical methods

Statistical analyses in Figures 1, 2, 4 and 5 were performed using GraphPad Prism 6 (GraphPad Software Inc.). Data in Figure 1c were analysed using the Kruskal–Wallis test followed by post‐tests comparing each group with HC. Data in Figures 2, 4 and 5 were log_2_‐normalised and analysed using a paired *t*‐test. In Figure 3, all data were then log_2_‐normalised for statistical analysis and principal component analysis, ANOVA, paired *t*‐tests, LEfSe and Random Forest analysis were performed using the Genomics Data Miner^21^. Heatmaps were generated using Heatmapper^77^.

## Author Contributions

**Simon H Apte:** Conceptualization; data curation; formal analysis; investigation; methodology; validation; writing–original draft; writing–review and editing. **Gabriela Minigo:** Conceptualization; data curation; formal analysis; investigation; methodology; validation; writing–original draft; writing–review and editing. **Penny L Groves:** Investigation. **Jessie C Spargo:** Investigation. **Magdalena Plebanski:** Conceptualization; investigation. **Mathew J Grigg:** Investigation; resources. **Enny Kenangalem:** Investigation; resources. **Julie G Burel:** Conceptualization. **Jessica R Loughland:** Investigation. **Katie L Flanagan:** Conceptualization, investigation. **Kim A Piera:** Investigation. **Timothy William:** Investigation; resources. **Ric N Price:** Investigation; funding acquisition; resources. **Tonia Woodberry:** Investigation. **Bridget E Barber:** Investigation; resources. **Nicholas M Anstey:** Conceptualization; funding acquisition; supervision; investigation; methodology; writing–original draft; writing–review and editing. **Denise L Doolan:** Conceptualization; funding acquisition; supervision; investigation; project administration; methodology; writing–original draft; writing–review and editing.

## Conflict of Interest

The authors declare no conflict of interest.
