# Supplementary material for: A population of CD4hiCD38hi T cells correlates with disease severity in patients with acute malaria
Source: Clin Transl Immunology. 2020 Nov 24;9(11):e1209. doi: 10.1002/cti2.1209 (PMC7684974; doi:10.1002/cti2.1209)
Supplement: Supplementary file 1 [file CTI2-9-e1209-s001.docx]

**
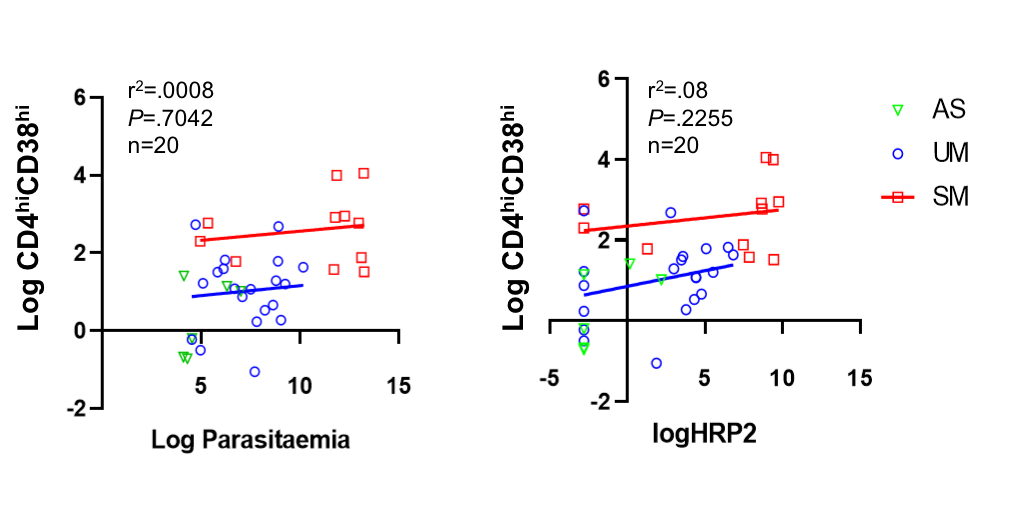
**

**Supplementary figure 1.** **Relationship between CD4^hi^CD38^hi^ cells and parasite burden.** Scatter plots of CD4^hi^CD38^hi^ frequency and (a) parasitaemia or (b) plasma HRP2 levels are shown for patients presented in Figure 1c. Pearson correlation of all parasitaemic patients shows a positive relationship (both r = 0.54; *P* = 0.0006). This relationship was not statistically significant when stratified by disease severity. Lines of best fit were determined by linear regression for data for UM (blue circles, blue line) and SM patients (red squares, red line) are shown. The number of samples correlated, rho-squared, and *P*-value are presented in each plot and correspond to correlations in UM patients. *P* < 0 .05 was considered significant.

**
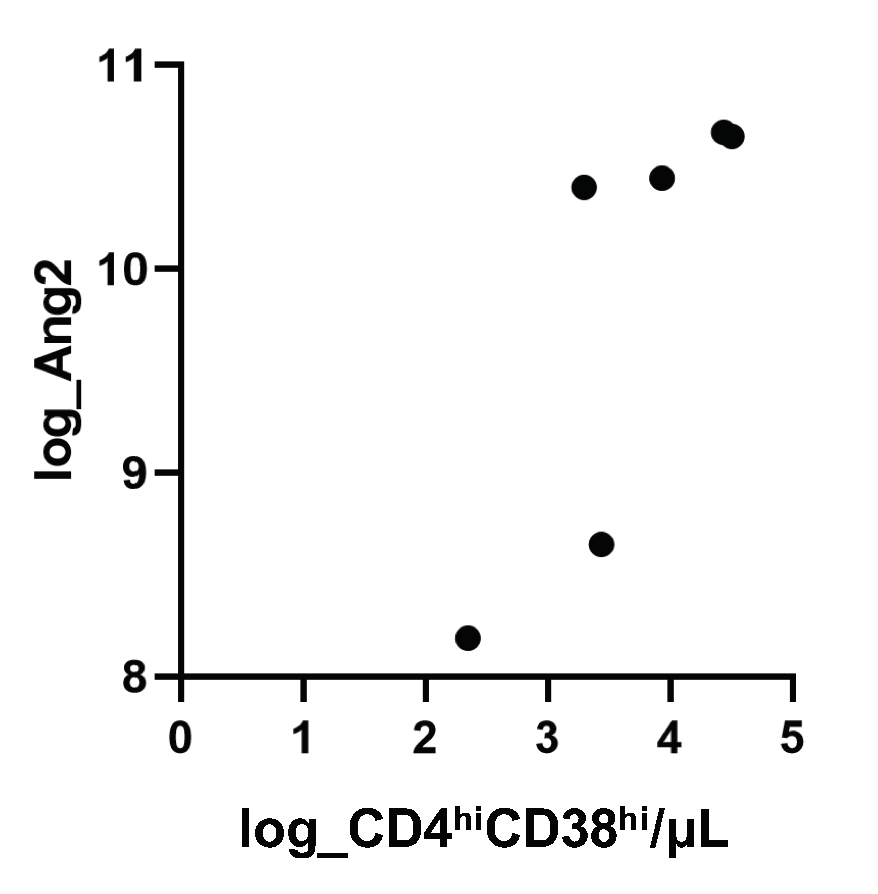
**

**Supplementary figure 2.** **Correlation of CD4^hi^CD38^hi^ counts and plasma Ang2 levels.** Lymphocyte counts were available for a subset of patients with SM (n=7) and absolute CD4^hi^CD38^hi^ counts calculated. Pearson correlation of absolute CD4^hi^CD38^hi^ counts and plasma Ang2 levels is shown (r = 0.825; *P*= 0.045).

| **Supplementary table 1. Wilcoxon signed-rank test comparing CD4^norm^ cells to CD4^hi^CD38^hi^ cells.** | | | | | | |
| --- | --- | --- | --- | --- | --- | --- |
| **Gene** | ***P*** | ***P*.adj** | **FDR qValue** | **Mean CD4^norm^** | **Mean CD4^hi^CD38^hi^** | **Fold Change** |
| LAG3 | 0 | 0 | 0 | 22 | 1398 | 62.22 |
| IL10 | 0 | 0 | 0 | 18 | 706 | 39.12 |
| HAVCR2 | 0 | 0 | 0 | 13 | 336 | 25.47 |
| CXCR6 | 0 | 0 | 0 | 16 | 113 | 7.02 |
| HLADRA | 0 | 0 | 0 | 25 | 155 | 6.09 |
| LGALS1 | 0 | 0 | 0 | 48 | 179 | 3.75 |
| JAK2 | 0 | 0 | 0 | 60 | 180 | 3.01 |
| LCK | 0 | 0 | 0 | 84 | 228 | 2.71 |
| CD3G | 0 | 0 | 0 | 196 | 471 | 2.40 |
| JAK1 | 0 | 0 | 0 | 190 | 315 | 1.65 |
| IL13 | 0 | 0 | 0 | 1291 | 181 | 0.14 |
| CD4 | 0 | 0.0001 | 0 | 18 | 120 | 6.84 |
| IL21 | 0 | 0.0001 | 0 | 393 | 2597 | 6.62 |
| MAF | 0 | 0.0001 | 0 | 177 | 547 | 3.10 |
| FASLG | 0 | 0.0001 | 0 | 180 | 62 | 0.34 |
| CCR4 | 0 | 0.0001 | 0 | 327 | 73 | 0.22 |
| ADORA2A | 0 | 0.0002 | 0 | 30 | 88 | 2.93 |
| NFATC2 | 0 | 0.0002 | 0 | 80 | 189 | 2.35 |
| PDCD1 | 0 | 0.0003 | 0 | 95 | 338 | 3.55 |
| IL22 | 0 | 0.0003 | 0 | 310 | 42 | 0.13 |
| IL2RB | 0 | 0.0005 | 0 | 115 | 268 | 2.33 |
| CD40LG | 0 | 0.0009 | 0.0001 | 1485 | 611 | 0.41 |
| CD27 | 0 | 0.001 | 0.0001 | 32 | 132 | 4.11 |
| GZMA | 0 | 0.0011 | 0.0001 | 15 | 100 | 6.53 |
| CXCL8 | 0 | 0.0015 | 0.0001 | 97 | 836 | 8.66 |
| PRDM1 | 0 | 0.0018 | 0.0001 | 151 | 328 | 2.17 |
| PVRIG | 0.0001 | 0.0034 | 0.0002 | 18 | 46 | 2.54 |
| IRF4 | 0.0001 | 0.0034 | 0.0002 | 1689 | 2823 | 1.67 |
| PRF1 | 0.0001 | 0.0037 | 0.0002 | 128 | 345 | 2.69 |
| ITGA4 | 0.0001 | 0.0054 | 0.0003 | 82 | 148 | 1.81 |
| TNF | 0.0003 | 0.012 | 0.0006 | 1960 | 1053 | 0.54 |
| S1PR1 | 0.0003 | 0.0155 | 0.0008 | 234 | 139 | 0.59 |
| CCL4 | 0.0004 | 0.017 | 0.0009 | 776 | 3595 | 4.63 |
| GZMH | 0.0004 | 0.0176 | 0.0009 | 22 | 51 | 2.32 |
| STAT3 | 0.0005 | 0.0229 | 0.0012 | 156 | 225 | 1.45 |
| CD226 | 0.0006 | 0.0267 | 0.0013 | 44 | 24 | 0.55 |
| IL18R1 | 0.0007 | 0.0298 | 0.0015 | 19 | 49 | 2.58 |
| JAK3 | 0.0008 | 0.0324 | 0.0016 | 144 | 231 | 1.60 |
| CD69 | 0.001 | 0.04 | 0.002 | 2262 | 1210 | 0.54 |
| STAT6 | 0.0022 | 0.0858 | 0.0043 | 67 | 100 | 1.48 |
| ICOS | 0.0042 | 0.1596 | 0.008 | 22 | 96 | 4.26 |
| FOXP3 | 0.0051 | 0.1887 | 0.0095 | 50 | 31 | 0.62 |
| CD3D | 0.0071 | 0.2556 | 0.0129 | 15 | 49 | 3.32 |
| STAT5B | 0.01 | 0.35 | 0.0177 | 80 | 127 | 1.60 |
| SELL | 0.011 | 0.374 | 0.0191 | 25 | 85 | 3.47 |
| RUNX1 | 0.012 | 0.396 | 0.0199 | 50 | 81 | 1.63 |
| KLF2 | 0.012 | 0.396 | 0.0199 | 417 | 305 | 0.73 |
| TNFRSF18 | 0.016 | 0.496 | 0.026 | 72 | 113 | 1.57 |
| FOS | 0.028 | 0.84 | 0.0446 | 383 | 139 | 0.36 |
| GZMB | 0.039 | 1 | 0.0596 | 108 | 198 | 1.83 |
| CCL5 | 0.039 | 1 | 0.0596 | 163 | 258 | 1.58 |
| IFNG | 0.042 | 1 | 0.0621 | 36 | 171 | 4.72 |
| CD28 | 0.043 | 1 | 0.0621 | 20 | 37 | 1.82 |
| CCR7 | 0.043 | 1 | 0.0621 | 62 | 39 | 0.62 |
| IL2RA | 0.044 | 1 | 0.0624 | 157 | 230 | 1.46 |
| KLRB1 | 0.051 | 1 | 0.071 | 18 | 41 | 2.31 |
| CXCR3 | 0.054 | 1 | 0.0739 | 44 | 76 | 1.75 |
| CD96 | 0.07 | 1 | 0.0941 | 217 | 188 | 0.87 |
| IL4R | 0.1 | 1 | 0.1322 | 142 | 105 | 0.74 |
| JUN | 0.16 | 1 | 0.208 | 87 | 66 | 0.76 |
| STAT4 | 0.17 | 1 | 0.2174 | 44 | 58 | 1.31 |
| CD45RA | 0.18 | 1 | 0.2265 | 58 | 44 | 0.76 |
| RUNX3 | 0.2 | 1 | 0.2438 | 212 | 244 | 1.15 |
| JUNB | 0.2 | 1 | 0.2438 | 792 | 706 | 0.89 |
| TBX21 | 0.21 | 1 | 0.252 | 75 | 90 | 1.20 |
| IL27RA | 0.27 | 1 | 0.3191 | 38 | 51 | 1.34 |
| GATA3 | 0.29 | 1 | 0.3376 | 186 | 148 | 0.80 |
| ITK | 0.44 | 1 | 0.5047 | 523 | 449 | 0.86 |
| PTGER2 | 0.46 | 1 | 0.52 | 23 | 19 | 0.85 |
| CXCR5 | 0.6 | 1 | 0.6686 | 44 | 49 | 1.13 |
| GNLY | 0.64 | 1 | 0.7031 | 29 | 26 | 0.89 |
| STAT5A | 0.65 | 1 | 0.7042 | 154 | 162 | 1.05 |
| IL7R | 0.72 | 1 | 0.7693 | 66 | 55 | 0.83 |
| IL21R | 0.76 | 1 | 0.8011 | 127 | 132 | 1.04 |
| BACH2 | 0.81 | 1 | 0.8424 | 110 | 115 | 1.04 |
| IL4 | 0.88 | 1 | 0.9032 | 315 | 304 | 0.96 |
| TGFB1 | 0.94 | 1 | 0.95 | 1607 | 1622 | 1.01 |
| STAT1 | 0.95 | 1 | 0.95 | 115 | 113 | 0.99 |

**Supplementary table 1. Genes detected in NanoStringTM analysis.** Genes reproducibly detected in NanoStringTM analysis were assessed by Wilcoxon signed-rank test comparing CD4^norm^ cells to CD4^hi^CD38^hi^ cells (Genomics Data Miner [21]). Genes were considered reproducibly detected if they were above the limit of detection in at least 50% of the samples.
